# High burden of variants of uncertain significance in early-onset colorectal cancer among indigenous African patients: a call for global research equity in cancer genetics

**DOI:** 10.1007/s11033-025-10750-6

**Published:** 2025-07-08

**Authors:** Safiye Yildiz, Ramadhani Chambuso, George Rebello, Raj Ramesar

**Affiliations:** UCT/MRC Genomic and Precision Medicine Research Unit, Division of Human Genetics, Department of Pathology, Institute of Infectious Disease and Molecular Medicine, University of Cape Town and Affiliated Hospitals, Cape Town, South Africa

**Keywords:** Early-onset colorectal cancer, Germline whole exome sequencing, Variant of uncertain significance, Pathogenic/likely pathogenic germline variants, Indigenous African populations, TCGA data

## Abstract

**Background:**

Colorectal cancer (CRC) remains a significant global health challenge, with rising incidence among early-onset cases in low- and middle-income countries, including South Africa. However, comprehensive germline genetic data from indigenous African populations remain scarce. This study aimed to explore germline genetic factors contributing to early-onset CRC (eoCRC) in Indigenous African patients using whole exome sequencing (WES).

**Methods and results:**

We performed WES on blood-derived genomic DNA from 32 Indigenous African patients diagnosed with eoCRC (< 50 years), who previously tested negative on a multigene CRC panel. While preliminary but definitive, pathogenic variants were identified in only 5 patients (16%) across genes such as *C6*,* FAT1*,* LZTR1*,* PYCR1*, and *UGT1A7*. A substantial proportion (47%, *n* = 15) carried variants of uncertain significance (VUS) with strong pathogenic potential (“leaning pathogenic”) in genes *ASXL1*,* CHEK2*,* ERBB2*,* ERCC4*,* INSR*,* KIT*,* MITF*,* NOTCH1*,* NOTCH2*,* PDGFRA*,* RAD51B*,* RAD54L*,* RASA1*,* RECQL*,* SUFU*,* VEGFA*, and *WT1*. Comparative analysis with public datasets and recurrent findings suggests these leaning pathogenic VUSs may represent true disease-associated variants, currently may be misclassified due to limited representation of African genomes in reference databases.

**Conclusions:**

Our findings reveal a high burden of potentially pathogenic VUSs in indigenous African patients with eoCRC, reflecting both unique genetic architecture and a critical gap in global genomic equity. These variants may contribute to future variant reclassification and improved understanding of CRC predisposition in African populations. This study underscores the urgent need for population-specific genomic research and the development of inclusive variant databases to support accurate diagnosis and personalised care.

**Supplementary Information:**

The online version contains supplementary material available at 10.1007/s11033-025-10750-6.

## Introduction

Colorectal cancer (CRC) represents a significant global health burden, ranking as the third most prevalent cancer and the second leading cause of cancer-related mortality [1]. The incidence of early-onset CRC (eoCRC) —affecting individuals under 50 years of age—has risen steadily, with approximately one in ten new CRC cases now occurring in younger individuals. However, the definition of “early-onset” varies, with thresholds ranging from 40 to 60 years depending on the population studied. In sub-Saharan Africa, a particularly concerning trend has emerged: the rising incidence of CRC, predominantly among individuals younger than 40 years [2, 3].

Despite this alarming increase, there is a significant gap in the understanding of the genetic underpinnings of eoCRC in indigenous African populations, which are underrepresented in genetic research. These populations are presenting with CRC at earlier ages and with more aggressive disease compared to non-African populations [3, 4]. This lack of comprehensive germline cancer genetics data poses a critical public health challenge, as effective screening, prevention, and targeted treatment strategies for eoCRC in these populations remain elusive without a better understanding of the underlying genetic factors.

In previous research on eoCRC in indigenous African patients from South Africa, we demonstrated that next-generation sequencing (NGS) of germline DNA using a 14-gene multigene panel (*APC*,* BMPR1A*,* EPCAM*,* MLH1*,* MSH2*,* MSH6*,* MUTYH*,* PMS2*,* POLD1*,* POLE*,* PTEN*,* SMAD4*,* STK11*, and *TP53*) significantly improved the identification of individuals at risk for inherited cancer syndromes, including Lynch syndrome [3]. Furthermore, we identified novel pathogenic/likely pathogenic germline variants (PGVs) in established CRC-related genes through this targeted approach.

The widespread use of whole exome sequencing (WES) is often hindered by its cost, data complexity, and the resource limitations of molecular diagnostics in low-income settings [5]. WES is thus, generally employed as a secondary approach, after screening for PGVs in well-characterised genes, particularly for conditions like CRC [6, 7]. To address the challenges associated with the high cost of WES, novel strategies are being developed in our laboratories. One example of these strategies is developing a scoring model to prioritise patients for the most appropriate and cost-effective genetic testing methods, before resorting to NGS [8].

In indigenous African populations, PGVs may diverge significantly from those found in non-African populations, potentially reflecting unique genetic and environmental factors influencing CRC risk. South Africa, located at the southernmost tip of the continent, presents a unique anthropological setting for studying the genetic factors underlying eoCRC in indigenous African populations. Migration patterns along the eastern and western coasts of Africa have historically converged in this region, creating a distinct genetic landscape [9]. These factors, combined with divergent environmental exposures, may contribute to the predisposition for eoCRC in this population [4, 8].

We hypothesise that germline WES in eoCRC patients from indigenous African populations will reveal a disproportionately high rate of variants of uncertain significance (VUS), many of which may lean toward pathogenicity and contribute to CRC predisposition. These “leaning pathogenic” variants are often overlooked or misclassified due to the limited representation of African genomes in existing reference databases, highlighting a critical gap in global genomic knowledge. By performing germline WES in indigenous African patients with unresolved molecular diagnoses despite prior targeted multigene panel testing, this study aims to not only identify potentially pathogenic variants but also to shed light on under-recognised disease-associated variation. Our findings may inform future reclassification efforts and underscore the importance of population-specific genomic research in improving hereditary cancer diagnostics and precision medicine strategies for African populations.

## Materials and methods

### Study cohort

**Fig. 1:**
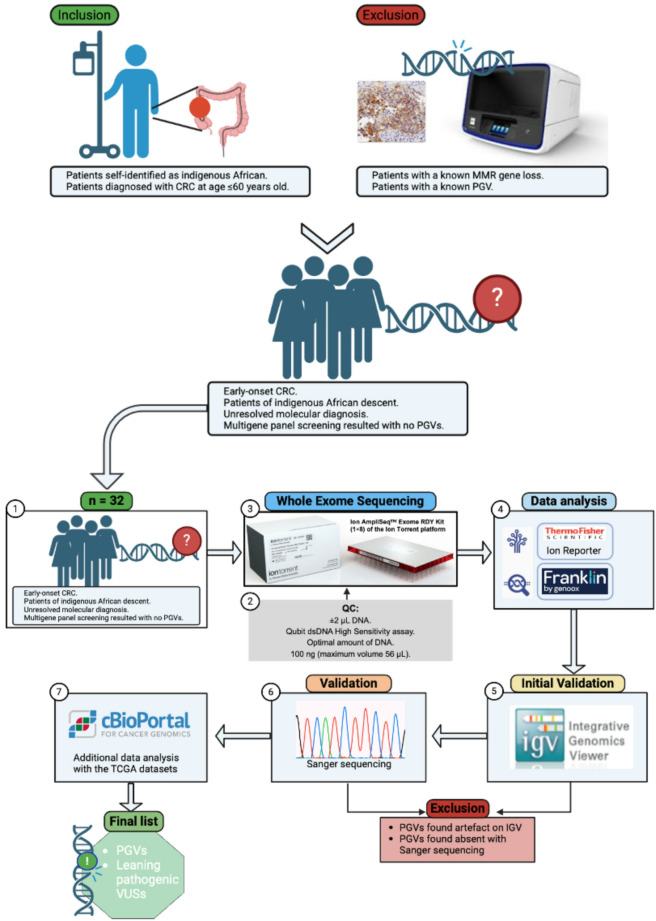
A flow diagram illustration of the whole methodology for patient selection and WES. Inclusion criteria (top left) involved patients recorded (self-identifying) as indigenous African and diagnosed with CRC at or before the age of 50 years. Exclusion criteria (top right). Number **(1)** Selection of 32 early-onset CRC, of indigenous African descent. Numbers **(2)**, and **(3)** DNA quality control (QC) included Qubit dsDNA High Sensitivity assay, and the library preparation. Number **(4)** Data analysis of the WES data on the Ion Reporter software and Genoox (Franklin) analysis for variant annotation. Numbers **(5)**, and **(6)** Initial validation of the prioritised variants on the Integrative Genomics Viewer (IGV) software followed by validation by Sanger sequencing. Number **(7)** Cross-referencing data analysis using available CRC datasets from the TCGA on the cBioPortal platform. Diagram created in BioRender (https://app.biorender.com/illustrations/6639ef2256418b3141e9f3d8?slideId=d0715115-29b8-4f5e- b145-bfb16ac28cce)

Participants in our cohort were from our CRC patient repository in the Division of Human Genetics, University of Cape Town, containing patients records from the Groote Schuur Hospital in Cape Town, and Chris Hani Baragwanath Hospital in Johannesburg. Patients were referred to as “indigenous African” based on their statements in the clinic for the self-identification criteria records when admitted to hospital (as required by the National Cancer Registry in this country where the cancer is a notifiable disorder). These patients were previously tested through a 14-gene NGS panel for hereditary CRC [3]. Patients who tested negative by the 14-gene panel were included in this cohort. The patients who were diagnosed with CRC at age equals or below 50 years whose molecular diagnosis were yet unknown, but clinically suggesting hereditary CRC due to early-onset of disease and clinical features of hereditary syndromes of CRC, e.g., family history, were included in the cohort (Fig. 1).

### Sample size and justification

Given the rarity of genetically unresolved eoCRC patients and the unique genetic background of the indigenous African populations, a sample size of 32 allowed for the identification of variants with a minor allele frequency (MAF) as low as 0.05 with reasonable confidence.

### Sample preparation and quality control

Samples with optimal DNA purity and integrity were selected for further preparation for WES. The required yield of DNA for manual library preparation was 100 ng to ensure an average sequencing depth of 100X is generated and other sequencing metrics for optimal quality of sequencing reads.

### Whole exome sequencing

Libraries were manually prepared using the Ion AmpliSeq™ Exome RDY Kit (1 × 8) of the Ion Torrent platform according to the manufacturer’s instructions (Applied Biosystems, Thermo Fisher Scientific, Massachusetts, USA). Ion Xpress™ Barcode Adapters were used for ligating the adapters to the amplicons. Barcoded libraries were purified using the Agencourt™ AMPure™ XP Reagent that contains magnetic beads allowing purification through bead suspension, according to the manufacturer’s instructions (Applied Biosystems, Thermo Fisher Scientific, Massachusetts, USA). The purified libraries were then eluted in low TE buffer. The concentration of each Ion AmpliSeq™ library was quantified by qPCR using the Ion Library TaqMan^®^ Quantitation Kit (Applied Biosystems, Thermo Fisher Scientific, Massachusetts, USA). Each diluted library (1:100) was then subject to qPCR on QuantStudio™ 3 Real- Time PCR System (Applied Biosystems, Thermo Fisher Scientific, Massachusetts, USA). Libraries were loaded onto the Ion 550™ Chips for templating on the Ion Chef instrument, using the Ion 550™ kit (Applied Biosystems, Thermo Fisher Scientific, Massachusetts, USA). Finally, sequencing was performed on an S5 Prime.

### Data processing and variant calling

The sequencing data were processed through the Ion Server software on the S5 Prime system (Applied Biosystems, Thermo Fisher Scientific, Massachusetts, USA), and the reads were aligned against the *Homo sapiens* reference genome 19 (hg19) to create *BAM* files. Variant calling and annotation were performed on the Ion Reporter software using a single DNA sample analysis workflow for germline DNA analysis. Variants were first called and annotated through Ion Reporter software. *VCF* files were generated and analysed per sample. Additionally, the *VCF* files generated by the S5 primer sequencer were then put through the Franklin annotation for further analysis of likely disease-causing variants to pick up any additional potential cancer associated variants. The output files were generated as *TSV* files (https://franklin.genoox.com/).

### Filtering and variant prioritisation

**Fig. 2:**
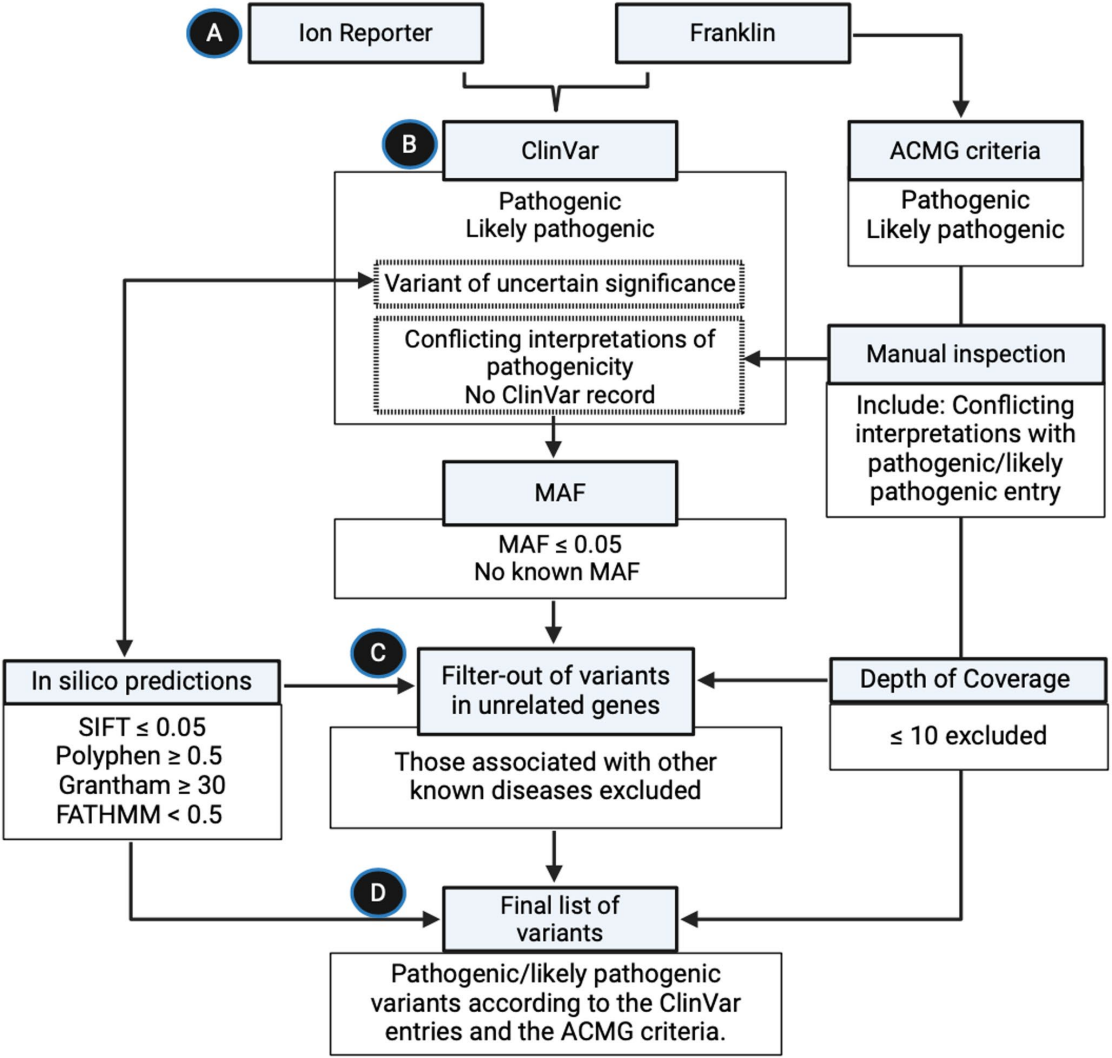
A workflow illustration for the filtering and classification of PGVs using multiple bioinformatics tools and criteria. **(A)** Genetic data are analysed using Ion Reporter and Franklin software, which filter variants through ClinVar for pathogenicity annotations. **(B)** VUS or those with conflicting interpretations are further assessed based on minor allele frequency (MAF) ≤ 0.05 or unknown MAF. **(C)** In-silico predictions using SIFT, Polyphen, Grantham, and FATHMM scores are applied to filter out benign variants. **(D)** Final list of variants incorporated the ACMG criteria and considers depth of coverage (≤ 10 reads). Variants in unrelated genes or associated with other known diseases were excluded, resulting in a final list of PGVs confirmed by ClinVar and ACMG criteria. “ACMG” = “American College of Medical Genetics and Genomics”, “MAF” = “minor allele frequency”

A rigorous variant filtering strategy was applied to prioritise those with potential pathogenicity in cancer, particularly hereditary CRC (Fig. 2). Variants marked as “pass” in Ion Reporter were considered, based on quality metrics such as sufficient read depth (≥ 10), minor allele frequency (MAF ≤ 0.05 or absent), and pathogenicity predictions from multiple tools. ClinVar annotations were also used to highlight known PGVs. The broader MAF threshold (up to 0.05) ensured rare, potentially population-specific variants were captured, addressing the limited representation of Indigenous African genomes in public databases [10].

For the Franklin output files, the filtering consisted of (i) ClinVar entry pathogenic/likely pathogenic, VUS or no entry; (ii) Franklin confidence of medium and high (the confidence calculated based on several parameters in the Franklin algorithm which includes the quality of the variant calling, strand bias fisher test, mapping quality, read position bias, total depth in the region, the base quality, and the allele balance); (iii) Franklin annotation being pathogenic, likely pathogenic, leaning pathogenic VUS (lpVUS). In addition, we considered the variants that had conflicting interpretations of pathogenicity in ClinVar and further inspected these manually to finalise their pathogenicity according to the ACMG criteria [6, 11]. The ClinVar conflicting interpretations were only considered if one of the entries was either a likely pathogenic, pathogenic, or VUS.

We then further inspected the filtered variants for pathogenicity. We used VarSome, Franklin, and InterVar to assist in classifying variants in accordance with ACMG guidelines [6, 11]. Because this cohort is unique and understudied, we preferred including any potential disease-causing variants in the final report. This involved: (i) each entry for each prioritised variants in ClinVar; we considered including variants in our final list if there is at least one pathogenic/likely pathogenic interpretation entry in ClinVar, (ii) variants that fulfil the ACMG criteria for pathogenicity, and (iii) we considered any gene that has been reported in a cancer related publication, in a pan cancer gene panel study or in literature as either cancer or hereditary cancer gene. This helped us extend the profile of potential disease-causing variants in an indigenous population group.

### Validation of the observed variants

Pathogenic or likely pathogenic variants that met our filtering criteria were selected for validation [12]. We visually inspected these variants in IGV, examining forward and reverse reads, neighbouring sequence context, and depth of coverage, flagging low-coverage calls (< 20%) or variants appearing across all samples as artefacts. This IGV step removed false-positives and validated the remaining variants, including VUSs identified by WES, before any further analyses.

The presence of the prioritised PGVs were validated using Sanger sequencing. Fresh aliquot of DNA samples was used for validation. Primers for these variants were designed and conditions for PCR amplification were optimised.

### VUSs and variants with conflicting interpretations of pathogenicity

The total list of VUSs from the Ion Reporter software (ClinVar) were merged for prioritisation of lpVUSs that involve strong evidence for pathogenicity [6]. To select those VUSs suggesting leaning pathogenic, we applied a similar filtering criteria for PGV prioritisation: we selected those (i) that have been recorded as VUS in ClinVar or without any entry in ClinVar; (ii) with either MAF ≤ 0.05 or no MAF record; (iii) with nonsynonymous function; (iv) with the following prediction scores, SIFT ≤ 0.05 (considered deleterious), Polyphen ≤ 0.5 (a high score leaning towards score 1 would have a greater likelihood of the variant being disease-causing), Grantham ≥ 30, and FATHMM ≤ 0.5 (suggesting a deleterious effect); (v) and those with information retrieved from the DRA database containing the keyword “neoplasm” to ensure cancer-associated VUSs would be prioritised. We then assessed these VUSs that suggested “leaning pathogenicity” using the Franklin platform. Variants that were classified by Franklin as “uncertain” but with a moderate to high likelihood of pathogenicity were designated as lpVUSs in this study.

### Comparison and data analysis using the cancer genome atlas program (TCGA) datasets on the cBioPortal for cancer genomics

For the potential PGVs and the lpVUSs prioritised after data analysis through Ion Reporter and Franklin we performed an additional data analysis by making use of the publicly available datasets from the Cancer Genome Atlas Program (TCGA) datasets on the cBioPortal site [13–15]. For this analysis, all the bowel cancer studies (*n* = 21) were selected and filtered for the selected genes prioritised in our study. The filtered data was inspected for the presence of the prioritised variants of interest in our study to observe whether any patient in the cBioPortal datasets carried the same variants. Co-occurrence of variants in two different genes provided in the cbioPortal were also analysed to obtain additional evidence for their disease relevance.

### Statistical analysis

The statistical analysis of this study was conducted using R software v4.3.0, to ensure rigorous and reproducible results [16]. Descriptive statistics were calculated to summarise patient demographics and sequencing metrics. Fisher’s exact test and chi-square tests were used to compare the frequency of variants between groups. Pearson’s correlation test was used to measure the strength of the linear relationship between two variables, and log-rank test was used for survival analysis. All tests were statistically significant at *p* < 0.05 with 95% confidence interval.

### Data availability

The datasets used and/or analysed during the current study are available from the corresponding author on reasonable request.

## Results

### Patient cohort and characteristics

Table 1 summarises the clinical and demographic characteristics of the CRC patients included in this study (*n* = 32). The mean age of disease onset was 34.2 years. Immunohistochemistry results were only available for 25% of the cohort, showing intact immunohistochemistry for the four major MMR genes. Among those where family history was reported, only 16% had a positive family history of cancer (Supplementary Table 1 for detailed patient information).

**Table 1:** Patient cohort and characteristics compared according to their presence of variant status.

Characteristics	Total cohort (N = 32)	Variant positive (n = 18)	Variant negative (n = 14)	*p*-value
**Diagnosis age (years)**				
Mean Age (SD)	34.2 (8.51)	35.5 (7.95)	32.5 (9.20)	0.332^**a**^
				(95% CI, -9.411 3.210)
Median age	33.5	36.5	30.5	0.2864
**Gender**				
Female	17 (53%)	11 (61.11%)	6 (42.86%)	0.5032^**b**^
Male	15 (47%)	7 (38.89%)	8 (57.14%)	
**Tumour localisation**				
Colon	19 (59.4%)	11 (63.16%)	8 (53.85%)	0.7241^**c**^
Rectum	12 (37.5%)	6 (31.58%)	6 (46.15%)	
Not available	1 (3.1%)	1 (5.56%)	0 (0.00%)	
**Tumour sidedness**				
Right	11 (34.37%)	5 (27.78%)	5 (35.71%)	0.7061^**c**^
Left	18 (56.25%)	11 (61.11%)	7 (50.00%)	
Both	2 (6.25%)	1 (5.56%)	1 (7.14%)	
Not available	1 (3.13%)	1 (5.56%)	1 (7.14%)	
**Family history of cancer**				
Yes	5 (15.63%)	5 (27.78%)	3 (21.43%)	0.5921^**c**^
No	8 (25%)	2 (11.11%)	3 (21.43%)	
Not available	19 (59.38%)	11 (63.16%)	8 (53.85%)	
**Immunohistochemistry**				
**status in MMR genes**				
All intact	8 (15.63%)	2 (11.11%)	3 (21.43%)	
Not available	24 (75%)	16 (84.21%)	8 (53.85%)	

^a^ t-test. ^b^ Chi-squared test. ^c^ Fisher’s exact test. Abbreviations: “SD” = “standard deviation. “CI” = confidence interval. Note: Variant positive patients are those who carry a PGV or a leaning pathogenic VUS after WES analysis

### Variant filtering outcomes after the WES analysis

**Fig. 3:**
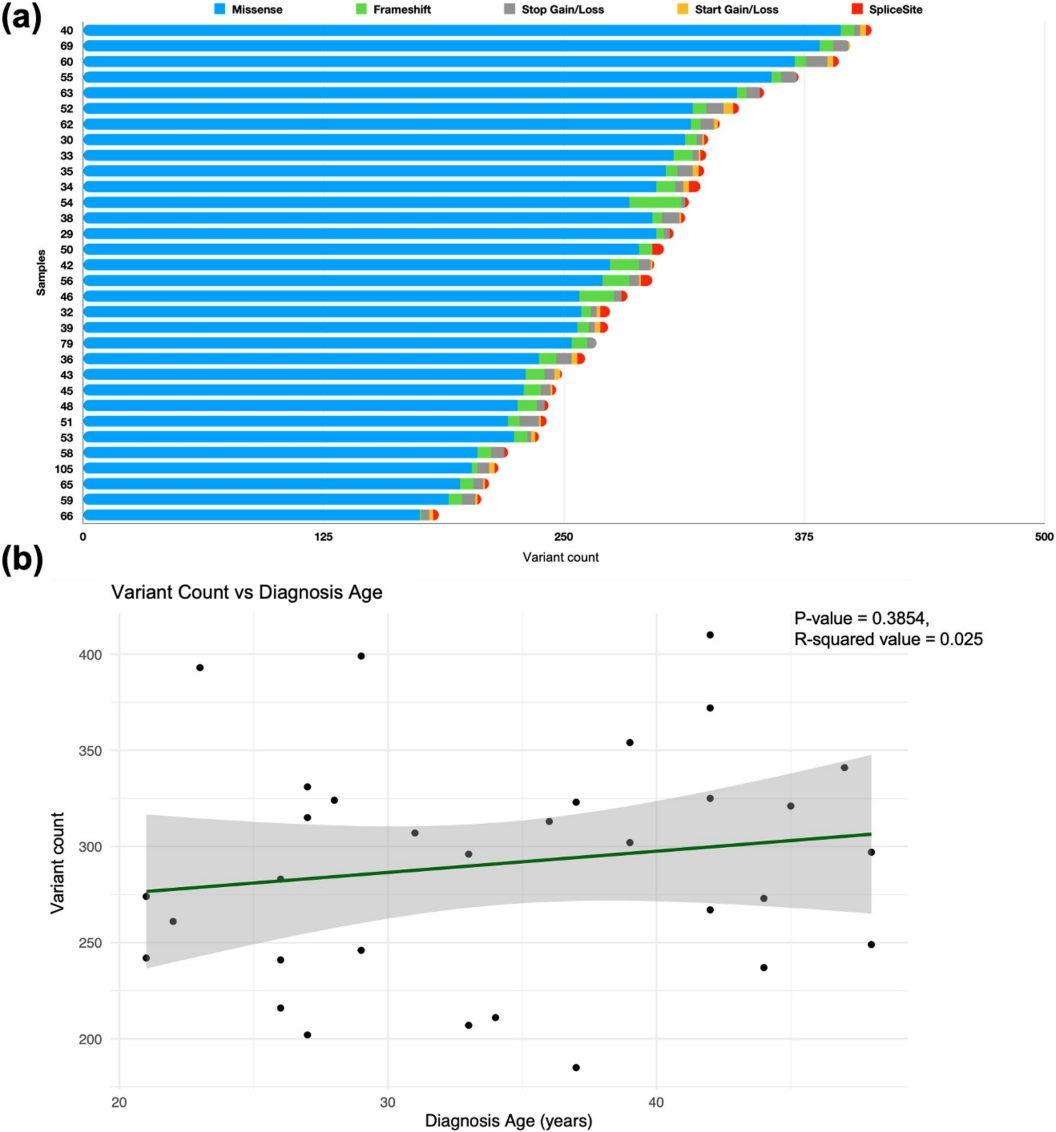
Variant count analysis. Variants with high quality, rare (MAF < 0.05), and classified as VUS or PGV. **(a)** Total variant count per patient in the WES cohort (*n* = 32). **(b)** Variant count per variant type, per patient

Variants with an MAF < 0.05, classified as VUS or PGV according to the ACMG criteria, with high or medium confidence provided by Franklin were extracted for variant count analysis for each patient and showed that the proportion of the missense variants was the highest, followed by frameshift, stop gain/loss, start gain/loss and splice site variants. Proportion of variants on each type of variant varied between patients (Fig. 3a). The relationship between variant count and the diagnosis age was not statistically significant (Fig. 3b).

### Variant prioritisation results after integrative genomics viewer inspection

Ninety-eight variants were initially prioritised for manual inspection on the IGV software that were PGVs based on the ClinVar entries or the ACMG criteria, after removal of duplicates. Visualising these variants on IGV, the numbers dropped to 14. The final list contained unique prioritised PGVs matching the ACMG criteria or being reported in the cancer-related literature (Supplementary Table 2).

### Clinical and genetic characteristics of patients with PGV or LpVUS

**Fig. 4:**
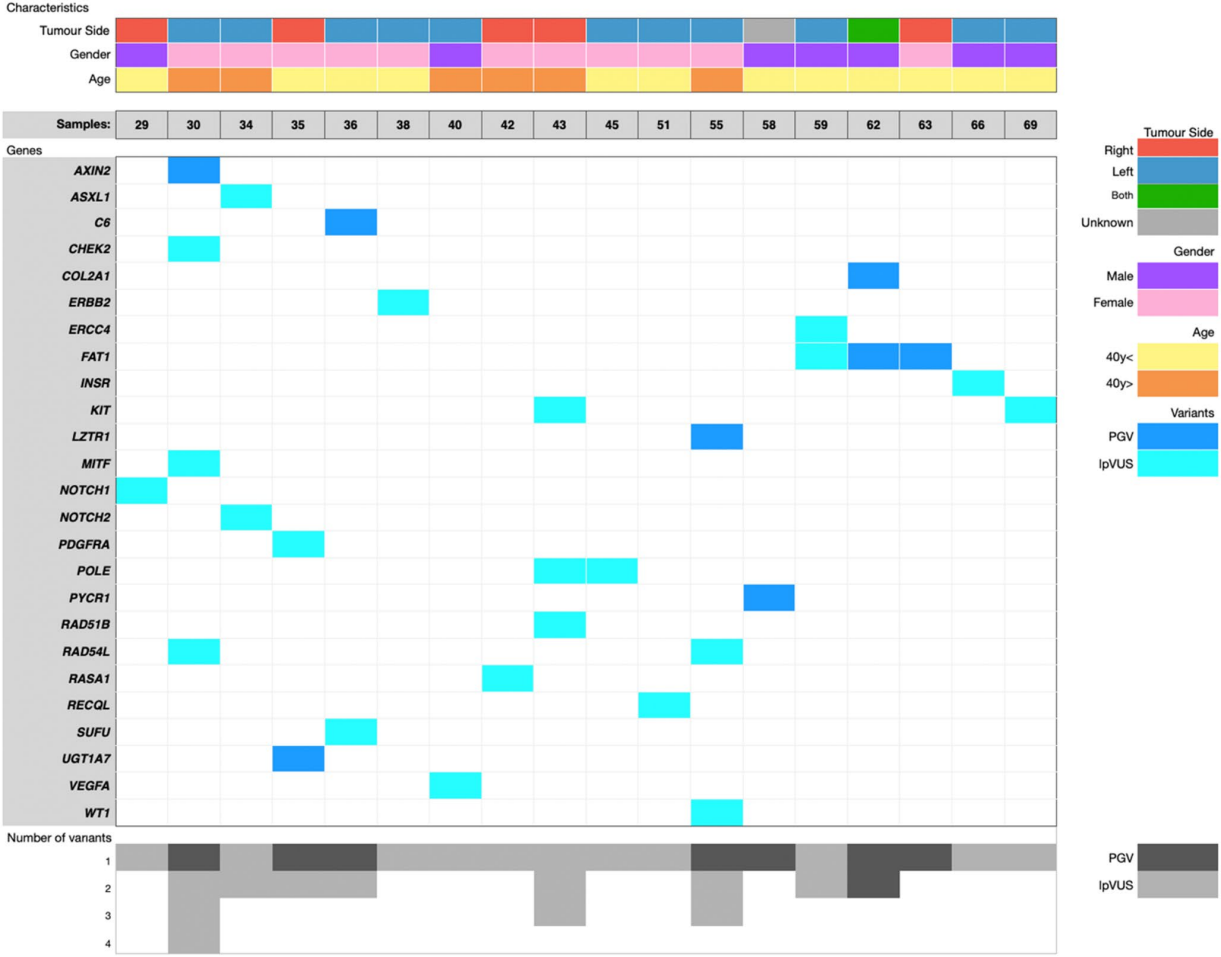
Correlation analysis between variant count and diagnosis age of patients in the WES cohort, from the highest total variant count to the lowest, while the diagnosis age varies among patients

Among patients who presented with a PGV and/or a lpVUS (*n* = 18), the majority were diagnosed at an age earlier than 40 years (*n* = 12, 67%), 61% had left-sided tumours, while 28% had right-sided tumours. Genetic analysis highlighted multiple variants across several genes, with notable recurrent variants in *FAT1*,* POLE*,* and RAD54L* (Fig. 4).

### Validation of WES- identified pathogenic variants by integrative genomics viewer inspection and Sanger sequencing

Seven patients presented with at least one PGV that could be associated with inherited CRC, prior to Sanger sequencing (Table 2). After validation, five patients were found to carry a PGV; among these, three patients had variants with at least one pathogenic/likely pathogenic entry in ClinVar. We summarised the details of these variants in Table 2 (see details on validations in Supplementary Fig. 1). The unconfirmed variants were excluded from further analyses.

**Table 2:** List of pathogenic/likely pathogenic germline variants prioritised in this study, after WES analysis

Sample ID	Variant	Locus	MAF	Protein	Function	Novel?	ACMG classification	ClinVar	Validated
**30**	*AXIN2*:c.1994delG	chr17:63532584	NA	p.Gly665Alafs*24	Frameshift	No	Pathogenic	Pathogenic	No
**35**	*UGT1A7*:c.243C > A	chr2:234590826	0.001	p.Tyr81Ter	Nonsense	No	Likely pathogenic	Uncertain	Yes
**36**	C6:c.821delA	chr5:41181566	0.001	p.Gln274ArgfsTer46	Frameshift	No	Pathogenic	Pathogenic	Yes
**52**	*ACTB*:c.323C > G	chr7:5568832	0.0	p.Ala108Gly	Missense	No	Likely pathogenic	None	No
**55**	*LZTR1*:c.1682G > A	chr22:21348913	NA	p.Arg561His	Missense	Yes	Leaning pathogenic VUS	Likely pathogenic	Yes
**58**	*PYCR1*:c.866A > G	chr17:79891184	NA	p.Lys289Arg	Missense	Yes	Leaning pathogenic VUS	Likely pathogenic entry	Yes
**62**	*FAT1*:c.5671C > A	chr4:187542069	0.001	p.Pro1891Thr	Missense	No	Leaning pathogenic VUS	Likely pathogenic entry	Yes
	*COL2A1*:c.917G > A	chr12:48387599	NA	p.Gly306Asp	Missense	Yes	Pathogenic	Pathogenic	No
**63**	*FAT1*:c.5671C > A	chr4:187542069	0.001	p.Pro1891Thr	Missense	No	Leaning pathogenic VUS	Likely pathogenic entry	Yes

Abbreviations: “MAF” = “minor allele frequency”, “ACMG” = “American College of Medical Genetics and Genomics”. “VUS” = Variant of uncertain significance”

### PGVs identified in the known cancer genes

We identified PGVs in cancer-related genes following the germline WES analysis. The *LZTR1* variant in patient 55 has been previously reported to associate with rare cancers affecting the nervous system such as schwannomas [17]. *LZTR1* is a gene involved in various cellular processes, including cell growth regulation and signalling pathways (e.g., chromatin remodeling pathway). It functions as a tumour suppressor gene, preventing uncontrolled cell proliferation. Patient 55 had intact MMR genes as confirmed by immunohistochemistry testing and was diagnosed with left-sided CRC at age 42 years. This patient also harboured a lpVUS in the *RAD54L* gene, which has been reported in a breast cancer patient from South Africa (Accession: RCV002226353.1, ClinVar).

Patient 35 was found with a likely pathogenic variant in *UGT1A7* gene, which is associated with CRC susceptibility, indicating a potential genetic predisposition in this patient. Patient 36 was found with a PGV in the *C6* gene. This variant has previously been reported in C6-related conditions such as certain autoimmune conditions, which are not directly associated with cancer but may promote tumour growth [18–20]. Patient 58 carried a likely pathogenic variant in *PYCR1*; however, this patient did not have substantial information on pathological features or family history of cancer but was diagnosed at a relatively younger age (of 27 years). Similarly, a likely pathogenic variant in the *FAT1* gene was identified in two patients, namely 62 and 63. This variant has been previously reported as likely pathogenic in a patient with Nephrotic syndrome, which is known to increase cancer risk by over 70% (Supplementary Table 3 for relevant pathway information [21]. It is important to note that the clinical records of these patients lacked information on the above-mentioned non-cancer syndromes within their families.

### Identification and validation of rare germline variants

A total of 494 rare VUSs (MAF ≤ 0.05) were identified across the cohort, including 126 in known hereditary cancer genes. After filtering with in-silico prediction tools (specifically, SIFT, Polyphen, Grantham, and FATHMM, as provided in the Ion Reporter software analysis) and confirming with IGV, 19 unique lpVUSs were prioritized (Table 3). These variants, located in exonic regions of cancer-related genes, may represent potential genetic risk factors for hereditary CRC. These prioritised lpVUS cases were identified in exonic regions of cancer-related genes and are predicted to be likely deleterious, providing valuable insights into potential genetic risk factors for hereditary CRC [11, 22, 23].

**Table 3:** List of prioritised VUS/leaning pathogenic VUS with potential pathogenicity in cancer-related genes

Sample ID	VUS/leaning pathogenic VUS	OncoKB (gene)	Locus	MAF	Protein
**29**	*NOTCH1:c*.1753G > A	Oncogenic, tumour suppressor.	chr9:139410085	NA	p.Ala585Thr
		T-Lymphoblastic Leukemia/Lymphoma			
		Chronic Lymphocytic Leukemia/Small Lymphocytic Lymphoma			
**30**	*CHEK2*:c.901T > A	Tumour suppressor.	chr22:29099500	NA	p.Leu301Met
		Breast, prostate and colorectal cancers			
	*MITF*:c.1520G > A	Oncogenic, tumour suppressor.	chr3:70014356	NA	p.Arg507Gln
		Melanoma and renal cell cancers.			
	*RAD54L*:c.2197T > G	Non-Hodgkin lymphoma and chronic lymphocytic leukemia.	Chr1:46743907	NA	p.Phe733Val
		Prostate cancer.			
		Breast cancer.			
**34**	*ASXL1*:c.3949C > A	Tumour suppressor. Myeloid malignancies.	chr20:31024464	NA	p.Pro1317Thr
	*NOTCH2*:c.3698C > T	Oncogenic, tumour suppressor.	chr1:120471793	NA	p.Pro1233Leu
		Hematologic malignancies.			
		Splenic Marginal Zone Lymphoma.			
**35**	*PDGFRA*:c.1850G > A	Oncogenic. Gastrointestinal stromal tumour.	chr4:55143618	NA	p.Arg617Gln
**36**	*SUFU*:c.1027C > T	Tumour suppressor. Pediatric medulloblastoma.	chr10:104375029	NA	p.Arg343Cys
**38**	*ERBB2*:c.1294C > T	Oncogenic. Breast, esophagogastric and endometrial cancers.	Chr17:37871770	NA	p.Arg432Trp
**40**	*VEGFA*:c.544delC	Oncogenic. Tumour initiation and angiogenesis. Overexpression in solid tumours.	chr6:43748585	0.0	p.His182IlefsTer39
**42**	*RASA1*:c.2470A > T	Tumour suppressor. Negative regulator of RAS, deleted or mutated in a number of cancers.	chr5:86674338	NA	p.Ser824Cys
**43**	*KIT*:c.1686G > C	Oncogenic. Gastrointestinal stromal tumours.	chr4:55593620	NA	p.Glu562Asp
	*POLE*:c.1004T > G	Tumour suppressor. POLE-related polyposis and colorectal cancer syndrome. Endometrial cancer.	chr12:133252696	NA	p.Phe335Cys
	*RAD51B*:c.914A > T	Tumour suppressor. Familial breast cancer.	chr14:68878201	NA	p.Asn305Ile
**45**	*POLE*:c.2672C > G	Tumour suppressor. POLE-related polyposis and colorectal cancer syndrome. Endometrial cancer.	chr12:133240624	NA	p.Ser891Cys
**51**	*RECQL*:c.809G > A	Tumour suppressor. Breast cancer susceptibility. Familial ovarian cancer, hereditary breast carcinoma.	chr12:21630795	0.0	p.Cys270Tyr
**55**	*WT1*:c.218A > T	Oncogenic, tumour suppressor.	chr11:32456689	NA	p.Gln73Leu
		Overexpression in various cancer types including leukemias.			
	*RAD54L*:c.460C > T	Non-Hodgkin lymphoma and chronic lymphocytic leukemia.	chr1:46726266	0.0	p.Arg154Trp
		Prostate cancer.			
		Breast cancer.			
**59**	*ERCC4*:c.1633G > A	Tumour suppressor. Xeroderma pigmentosum. predisposition to certain cancers.	chr16:14029422	NA	p.Gly545Arg
	*FAT1*:c.11224G > T	Tumour suppressor. Inactivation in various cancer types.	chr4:187524456	0.0	p.Asp3742Tyr
**66**	*INSR*:c.320C > T	Oncogenic. Altered by chromosomal rearrangement in colorectal cancers.	chr19:7267688	NA	p.Thr107Met
**69**	*KIT*:c.1686G > C	Oncogenic. Gastrointestinal stromal tumours.	chr4: 55593620	NA	p.Glu562Asp

Abbreviations: “MAF” = “minor allele frequency”, “ACMG” = “American College of Medical Genetics and Genomics”. “VUS” = “Variant of uncertain significance”. "OncoKB" = "MSK's Precision Oncology Knowledge Base"

### Cross-reference of germline variants with somatic data and variant frequency from the TCGA

To provide further evidence for the potential pathogenicity of the PGVs and lpVUSs identified in this study, we analysed available CRC data from patients in the TCGA PanCancer Atlas on the cBioPortal cancer genomics database (https://www.cbioportal.org/). Firstly, the 21 datasets for bowel cancer were scanned for these variants in CRC patients (Supplementary Table 4, for the full list of the studies selected for this analysis). This analysis showed that none of the PGV or lpVUS cases identified in our study were present in the cBioPortal bowel cancer datasets with somatic data. Secondly, we then browsed the other cancer types in the TCGA PanCancer Atlas datasets (Supplementary Tables 5,** 32** PanCancer studies). We found that the *LZTR1* variant was reported in one patient with kidney renal papillary cell carcinoma in the TCGA PanCancer Atlas. Among the lpVUSs, *MITF* was present in one patient with CRC, more specifically rectal adenocarcinoma. The *NOTCH1* germline variant that was present in one of our patients (patient 29), was also found in somatic variants in the TCGA PanCancer Atlas, in an individual affected with pancreatic cancer (Supplementary Table 4).

Additionally, it is important to note that the TCGA PanCancer Atlas datasets had patients with a PGV in these genes. Although the TCGA data are somatic, these PGVs were previously reported in germline cases in ClinVar. These genes with a PGV as reported in affected individuals with various cancers previously (ClinVar, germline) included: *ASXL1*,* CHEK2*,* KIT*,* MITF*,* NOTCH1*,* PDGFRA*,* POLE*,* RAD54L*,* SUFU*, and *WT1* (Supplementary Table 6*). RAD51B* with no PGVs but a lpVUS reported in germline in hereditary breast ovarian cancer syndrome.

Variant frequencies in *ASXL1* and *MITF* was the second highest in CRC, while *ERCC4* and *POLE* were third highest, and *INSR*, *KIT*, *PDGFRA*, *RAD54L*, and *RECQL* were the fourth highest (Supplementary Fig. 2a). In comparison with other cancer types in the TCGA studies: In endometrial cancers, *ASXL1*, *CHEK2*,* ERCC4*,* INSR*,* MITF*,* NOTCH2*,* POLE*,* RAD51B*,* RAD54L*,* RASA1*,* RECQL*, and *SUFU* had the highest variant frequencies. The variant frequency of *ERBB2* was third highest in endometrial cancer, seventh in CRC, and eighth in breast cancer. The variant frequency for *WT1* was fourth in endometrial cancer and sixth in CRC, while *FAT1* was second in endometrial cancer and seventh in CRC. These findings highlight the significant variant frequencies of our genes of interest across various cancer types, reinforcing their potential roles in cancer pathogenesis (Supplementary Fig. 2b for the remaining of 19 genes).

#### Mutual exclusivity and co-occurrence of variants in different genes

Finally, we aimed to investigate the mutual exclusivity and co-occurrence of variants in different genes among patients identified with lpVUSs in the TCGA datasets. This analysis focused on cases where lpVUSs were present in multiple genes within our cohort (patients 30, 34, 43, 55, and 59). This approach provided additional evidence supporting the pathogenicity of these identified lpVUSs in CRC predisposition. Specifically, patient 30 had lpVUSs in *CHEK2*, *MITF* and *RAD54L* genes. TCGA datasets revealed significant co-occurrence of variants between *CHEK2* and *RAD54L*, *CHEK2* and *MITF*, and *MITF* and *RAD54L* (p-value < 0.001). Patient 34 had a lpVUSs in *ASXL1* and *NOTCH2 genes*, with significant co-occurrence of variants in these genes (p-value < 0.001) (Supplementary Table 7). Moreover, the overall survival analysis of patients in the selected TCGA PanCancer Atlas datasets indicated that the altered group (patients with variants in the 19-genes containing a lpVUS) containing 2387 patients (22% of the total patients) had significantly better survival compared to the unaltered group, log-rank test p-value of 0.0114 (Supplementary Fig. 3).

The datasets were further selected for the unaltered 14-known genes of hereditary CRC (*APC*,* BMPR1A*,* EPCAM*,* MLH1*,* MSH2*,* MSH6*,* MUTYH*,* PMS2*,* POLD1*,* POLE*,* PTEN*,* SMAD4*,* STK11*, and *TP53*) but with altered genes of the lpVUS cases that were identified in this study to observe their clinical or mutational features. This analysis showed that the median age in this group was 60 years (*n* = 553 patients, Supplementary Fig. 4a, Supplementary Table 5). The earliest diagnosis age was in the *KIT* and *VEGFA*-mutated groups (Supplementary Fig. 4b). Furthermore, it showed that only patients with *FAT1* variants had CRC, and most patients harboured variants in genes such as *CHEK2*, *ERBB2*, and *KIT* in most of the cancers (Supplementary Fig. 4c). We also observed that patients with variants in *ASXL1*,* FAT1*,* NOTCH2*,* RECQL*,* CHEK2*,* ERBB2*,* INSR*,* NOTCH1*,* PDGFRA*,* RASA1* and *WT1* also harboured variants in *BRCA1/2* genes (Supplementary Fig. 4d).

## Discussion

We present a comprehensive germline WES analysis of 32 genetically unresolved eoCRC patients from an indigenous African population in South Africa. Notably, no PGVs were detected in well-known hereditary CRC genes, despite the early onset of disease. Identified variants in genes such as *UGT1A7*, *PYCR1*, *LZTR1*, and *FAT1*, which are involved in metabolic pathways, chemical carcinogenesis, MAPK signalling, and Wnt signalling, point to novel contributors to CRC in this understudied population [24–27]. This reinforces the hypothesis that eoCRC in this population may be driven by novel or under-characterised genetic factors not captured by traditional panels, underscoring the limitations of applying Eurocentric genetic frameworks to underrepresented populations [28].

A striking finding was the high proportion of VUSs with strong evidence leaning toward pathogenicity—referred to as “leaning pathogenic” variants. These variants were found in over 45% of the cohort and occurred in genes with known relevance to cancer biology, including *CHEK2*,* RAD54L*,* NOTCH2*,* ASXL1*,* PDGFRA*, and *KIT* [29–35]. Although these genes are not traditionally associated with CRC predisposition, their involvement in key oncogenic pathways and in other hereditary cancer syndromes suggests a potential contributory role in CRC pathogenesis within this population.

Genes such as *SUFU*, which harboured an lpVUS in one of the patients diagnosed at 22 years of age, is known for its association with Gorlin syndrome, while *ASXL1* and *NOTCH2*, identified in another patient, have been implicated in familial CRC [31, 32, 36]. The presence of multiple lpVUSs in some individuals—such as patient 30, who carried variants in *CHEK2*,* MITF*, and *RAD54L*—raises the possibility of oligogenic contributions to disease, which could explain rapid disease progression in the absence of classical PGVs [37, 38]. Similarly, *RECQL*, a gene associated with breast/ovarian cancer, was found in a patient diagnosed with rectal cancer at 26 years old, underscoring the need to explore co-occurring cancer syndromes in this population [35]. Variants in *KIT* and *PDGFRA* also point to a possible link between familial gastrointestinal stromal tumours and CRC [39, 40]. These findings suggest that multigene panels for hereditary CRC should be expanded to include genes beyond the traditional hereditary CRC syndromes, particularly for underrepresented populations [41].

This study identifies lpVUSs in genes not typically linked to CRC, highlighting their potential role in disease predisposition. The broader gene analysis was driven by the possibility of misclassified primary tumours and increasing evidence that genes associated with other cancers (e.g., *BRCA1*,* BRCA2*,* CHEK2*) may also influence CRC risk through shared pathways. Given the scarcity of genomic data from indigenous African populations, this approach offers important insight into under-recognised genetic contributors to CRC in these communities. The distribution of PGVs and lpVUSs across our cohort underscores that a one-size-fits-all approach may not adequately address the diverse genetic risks in CRC patients [42, 43]. Functional studies are necessary to clarify the roles of VUSs, particularly lpVUSs, in cancer-related genes, which could significantly impact patient management and reclassification of these variants [44].

The enrichment of such variants in our cohort also reflects a broader issue in global genomics: the severe underrepresentation of African populations in public variant databases [45, 46]. This gap leads to misclassification of clinically significant variants and impedes the development of accurate, equitable diagnostic tools. Our findings support prior literature suggesting that African genomes harbour unique, potentially impactful variants that remain largely uncharacterised [46].

Our findings suggest that these populations harbour distinct genetic variants that could reveal novel pathways and mechanisms contributing to CRC [47]. The absence of known hereditary CRC gene variants contrasts with findings in other populations, suggesting that comprehensive genomic and epigenomic analyses are needed to uncover population-specific genetic risks [3, 48].

Clinically, the absence of PGVs in known CRC genes, coupled with the high burden of “leaning pathogenic” VUSs, challenges the sufficiency of current CRC multigene panels for African populations. There is a clear need to expand these panels to include cancer-related genes with emerging evidence of relevance in non-European groups. Furthermore, integrative approaches using public databases and somatic data—as done in this study—should be incorporated routinely to better interpret uncertain variants, especially in poorly studied populations [49]. Alternatively, environmental or microbial factors may also contribute to eoCRC. For instance, emerging evidence suggests a link between eoCRC and specific mutational signatures (e.g., SBS88 associated with ID18), potentially caused by early-life exposure to colibactin-producing bacteria. Investigating the prevalence of such signatures in tumour DNA from indigenous African patients represents a promising direction for future research [50].

Importantly, our comparative analysis with TCGA PanCancer Atlas data further supported the pathogenic potential of several variants, revealing overlap with somatic variants found in CRC and other cancers. These overlaps may help inform future reclassification efforts and suggest that some germline VUSs could have dual roles in somatic tumorigenesis [13–15].

### Limitations and contextual considerations

A paired germline–somatic WES analysis would have further strengthened the interpretation of the observed variants in relation to CRC predisposition. However, our study population is from an under-researched group, and tumour tissue collection was either not systematically performed at the time of diagnosis or the available specimens were too old to be retrieved from hospital repositories. Additionally, neither tumour samples nor fresh blood samples were available in long-term biobanks.

Family history is another important factor in evaluating hereditary cancer risk. Unfortunately, structured documentation of family cancer history has historically been lacking in the local healthcare system due to limited clinical infrastructure and the absence of formalised protocols for collecting this information during initial patient encounters. This area is currently undergoing substantial improvement nationally, as awareness of the importance of familial cancer risk and genetic research grows.

In this context, our study contributes uniquely by focusing on an indigenous African cohort, and to our knowledge, this is the first WES analysis performed on such a population group with early-onset CRC.

Importantly, the identification of a high burden of VUSs—especially those with bioinformatic evidence leaning toward pathogenicity—is not considered a limitation but a key finding. These “unknowns” reflect the paucity of genomic data from African populations and reveal potentially novel, unreported variants. Expanding such research across additional African cohorts and integrating findings into global genomic databases is essential. Our results underscore the urgent need for population-specific cancer genetics research to address global health inequities and represent a foundational step toward a more inclusive, equitable approach in precision oncology [12].

### Recommendations for future clinical and research practices in South Africa

Based on the findings and limitations of this study, we propose the following recommendations to improve genetic research and diagnostics for eoCRC in South Africa, particularly among indigenous African populations:

(i) Comprehensive genetic profiling of early-onset, MMR-proficient CRC cases should be prioritised to uncover novel germline contributions beyond known hereditary syndromes.
(ii) Expanded evaluation of candidate genes, including those highlighted in this study, is needed to better understand their potential roles in colorectal carcinogenesis within this unique population. A considerable effort ought to be expended in e.g., WES or even whole genome sequencing, with parallel RNA sequencing studies to more fully gauge the impact of previously undescribed genes in cancers of underrepresented populations.
(iii) The impact of early-life exposure to colibactin-producing bacteria, and its associated mutational signatures (e.g., SBS88/ID18), should be investigated in indigenous African cohorts as a potential contributor to eoCRC development.
(iv) Tumour tissue collection and preservation should be systematically implemented in future patient recruitments to enable paired germline-somatic analysis, including LOH and mutational profiling.
(v) Standardised family history documentation, including the implementation of structured and culturally adapted questionnaires at the time of diagnosis, together with comprehensive clinical records, must be integrated into clinical practice to support hereditary cancer risk assessment and facilitate cascade genetic testing.

These steps are essential to building a sustainable, equitable genomic research infrastructure in South Africa and to addressing longstanding disparities in cancer genomics research across African populations.

## Conclusion

This study addresses the significant underrepresentation of African populations in germline genomic research by presenting WES data from eoCRC patients of indigenous African ancestry in South Africa. Despite the absence of PGVs in well-known hereditary CRC genes, we identified a high rate of rare and previously unreported VUSs, many of which lean toward pathogenicity based on bioinformatic evidence and functional relevance to cancer pathways.

These findings underscore the distinct genetic architecture of CRC in this understudied population and the urgent need to improve diversity in genetic reference databases. By prioritising and characterising these “leaning pathogenic” VUSs, our study contributes valuable preliminary insights that may guide future variant reclassification, inform population-specific gene panel development, and advance more equitable genomic medicine. This work highlights the strength of leveraging WES to uncover novel candidate variants in populations historically excluded from genomic studies and lays critical groundwork for improving CRC risk assessment and personalised care in African settings.

## Electronic supplementary material

Below is the link to the electronic supplementary material.

Supplementary Material 1

Supplementary Material 2

Supplementary Material 3

Supplementary Material 4

## Data Availability

The datasets used and/or analysed during the current study are available from the corresponding author on reasonable request.

## Abbreviations

ACMG: American College of Medical Genetics and Genomics
CRC: Colorectal cancer
IGV: Integrative Genomics Viewer
lpVUS: Leaning pathogenic variant of uncertain significance
MAF: Minor allele frequency
NGS: Next-generation sequencing
PGV: Pathogenic/likely pathogenic germline variant
TCGA: The Cancer Genome Atlas Program
UCT: University of Cape Town
VUS: Variant of uncertain significance
WES: Whole Exome Sequencing
