# Supplementary material for: High burden of variants of uncertain significance in early-onset colorectal cancer among indigenous African patients: a call for global research equity in cancer genetics
Source: Mol Biol Rep. 2025 Jul 8;52(1):684. doi: 10.1007/s11033-025-10750-6 (PMC12238126; doi:10.1007/s11033-025-10750-6)
Supplement: Supplementary file 1 — Supplementary Material 1 [file 11033_2025_10750_MOESM1_ESM.pdf]

High Burden of Variants of Uncertain Significance in Early-Onset Colorectal Cancer Among Indigenous African Patients: A Call for Global Research Equity in Cancer Genetics

Molecular Biology Reports
Safiye Yildiz[1], Ramadhani Chambuso[1], George Rebello[1], and Raj Ramesar[1*]

[1]UCT/MRC Genomic and Precision Medicine Research Unit, Division of Human Genetics, Department of Pathology, Institute of Infectious Disease and Molecular Medicine, University of Cape Town and Affiliated Hospitals, Cape Town, South Africa.

*Raj Ramesar**. Email address:** raj.ramesar@uct.ac.za

Supplementary Table 5: The full list of 32 PanCancer studies on the CBio Portal.

Selected Studies

Deselect all

Adrenal Gland

Adrenocortical Carcinoma

- [x] Adrenocortical Carcinoma (TCGA, PanCancer Atlas) — 92 samples

Biliary Tract

Intraductal Papillary Neoplasm of the Bile Duct

→ CHOLANGIOCARCINOMA

- [x] Cholangiocarcinoma (TCGA, PanCancer Atlas) — 36 samples

Bladder/Urinary Tract

Bladder Urothelial Carcinoma

- [x] Bladder Urothelial Carcinoma (TCGA, PanCancer Atlas) — 411 samples

Bowel

Colorectal Adenocarcinoma

- [x] Colorectal Adenocarcinoma (TCGA, PanCancer Atlas) — 594 samples

Breast

Invasive Breast Carcinoma

- [x] Breast Invasive Carcinoma (TCGA, PanCancer Atlas) — 1084 samples

CNS/Brain

Diffuse Glioma

- [x] Brain Lower Grade Glioma (TCGA, PanCancer Atlas) — 514 samples

→ GLIOBLASTOMA

- [x] Glioblastoma Multiforme (TCGA, PanCancer Atlas) — 592 samples

Cervix

Cervical Squamous Cell Carcinoma

- [x] Cervical Squamous Cell Carcinoma (TCGA, PanCancer Atlas) — 297 samples

Esophagus/Stomach

Esophagogastric Adenocarcinoma

→ ESOPHAGEAL ADENOCARCINOMA

- [x] Esophageal Adenocarcinoma (TCGA, PanCancer Atlas) — 182 samples

→ STOMACH ADENOCARCINOMA

- [x] Stomach Adenocarcinoma (TCGA, PanCancer Atlas) — 440 samples

Eye

Ocular Melanoma

→ UVEAL MELANOMA

- [x] Uveal Melanoma (TCGA, PanCancer Atlas) — 80 samples

Head and Neck

Head and Neck Squamous Cell Carcinoma DESELECT ALL

- [x] Head and Neck Squamous Cell Carcinoma (TCGA, PanCan… — 523 samples

Kidney

Renal Cell Carcinoma

→ **RENAL CLEAR CELL CARCINOMA**

- [x] Kidney Renal Clear Cell Carcinoma (TCGA, PanCancer Atlas) — 512 samples

→ **RENAL NON-CLEAR CELL CARCINOMA**

- [x] Kidney Chromophobe (TCGA, PanCancer Atlas) — 65 samples
- [x] Kidney Renal Papillary Cell Carcinoma (TCGA, PanCancer A… — 283 samples

Liver

Hepatocellular Carcinoma

- [x] Liver Hepatocellular Carcinoma (TCGA, PanCancer Atlas) — 372 samples

Lung

Non-Small Cell Lung Cancer

→ **LUNG ADENOCARCINOMA**

- [x] Lung Adenocarcinoma (TCGA, PanCancer Atlas) — 566 samples

→ **LUNG SQUAMOUS CELL CARCINOMA**

- [x] Lung Squamous Cell Carcinoma (TCGA, PanCancer Atlas) — 487 samples

Lymphoid

Lymphoid Neoplasm

→ **NON-HODGKIN LYMPHOMA**

- [x] Diffuse Large B-Cell Lymphoma (TCGA, PanCancer Atlas) — 48 samples

Myeloid DESELECT ALL

Myeloid Neoplasm

→ **ACUTE MYELOID LEUKEMIA**

- [x] Acute Myeloid Leukemia (TCGA, PanCancer Atlas) — 200 samples

Ovary/Fallopian Tube

Ovarian Epithelial Tumor

→ SEROUS OVARIAN CANCER

- [x] Ovarian Serous Cystadenocarcinoma (TCGA, PanCancer Atl… — 585 samples

Pancreas

Pancreatic Adenocarcinoma

- [x] Pancreatic Adenocarcinoma (TCGA, PanCancer Atlas) — 184 samples

Pleura

Pleural Mesothelioma

- [x] Mesothelioma (TCGA, PanCancer Atlas) — 87 samples

Prostate

Prostate Adenocarcinoma

- [x] Prostate Adenocarcinoma (TCGA, PanCancer Atlas) — 494 samples

Skin

Melanoma

→ CUTANEOUS MELANOMA

- [x] Skin Cutaneous Melanoma (TCGA, PanCancer Atlas) — 448 samples

Soft Tissue

- [x] Pheochromocytoma and Paraganglioma (TCGA, PanCancer… — 178 samples
- [x] Sarcoma (TCGA, PanCancer Atlas) — 255 samples

Testis

- [x] Testicular Germ Cell Tumors (TCGA, PanCancer Atlas) — 149 samples

Thymus

Thymic Epithelial Tumor

→ THYMOMA

- [x] Thymoma (TCGA, PanCancer Atlas) — 123 samples

Thyroid

Well-Differentiated Thyroid Cancer

→ PAPILLARY THYROID CANCER

- [x] Thyroid Carcinoma (TCGA, PanCancer Atlas) — 500 samples

Uterus

Endometrial Carcinoma

- [x] Uterine Corpus Endometrial Carcinoma (TCGA, PanCancer … — 529 samples

→ UTERINE CARCINOSARCOMA/UTERINE MALIGNANT MIXED MULLERIAN TUMOR

- [x] Uterine Carcinosarcoma (TCGA, PanCancer Atlas) — 57 samples
