# Supplementary material for: High burden of variants of uncertain significance in early-onset colorectal cancer among indigenous African patients: a call for global research equity in cancer genetics
Source: Mol Biol Rep. 2025 Jul 8;52(1):684. doi: 10.1007/s11033-025-10750-6 (PMC12238126; doi:10.1007/s11033-025-10750-6)
Supplement: Supplementary file 2 — Supplementary Material 2 [file 11033_2025_10750_MOESM2_ESM.pdf]

High Burden of Variants of Uncertain Significance in Early-Onset Colorectal Cancer Among Indigenous African Patients: A Call for Global Research Equity in Cancer Genetics

Molecular Biology Reports

Safiye Yildiz[1], Ramadhani Chambuso[1], George Rebello[1], and Raj Ramesar[1*]

[1]UCT/MRC Genomic and Precision Medicine Research Unit, Division of Human Genetics, Department of Pathology, Institute of Infectious Disease and Molecular Medicine, University of Cape Town and Affiliated Hospitals, Cape Town, South Africa.

*Raj Ramesar**. Email address:** raj.ramesar@uct.ac.za

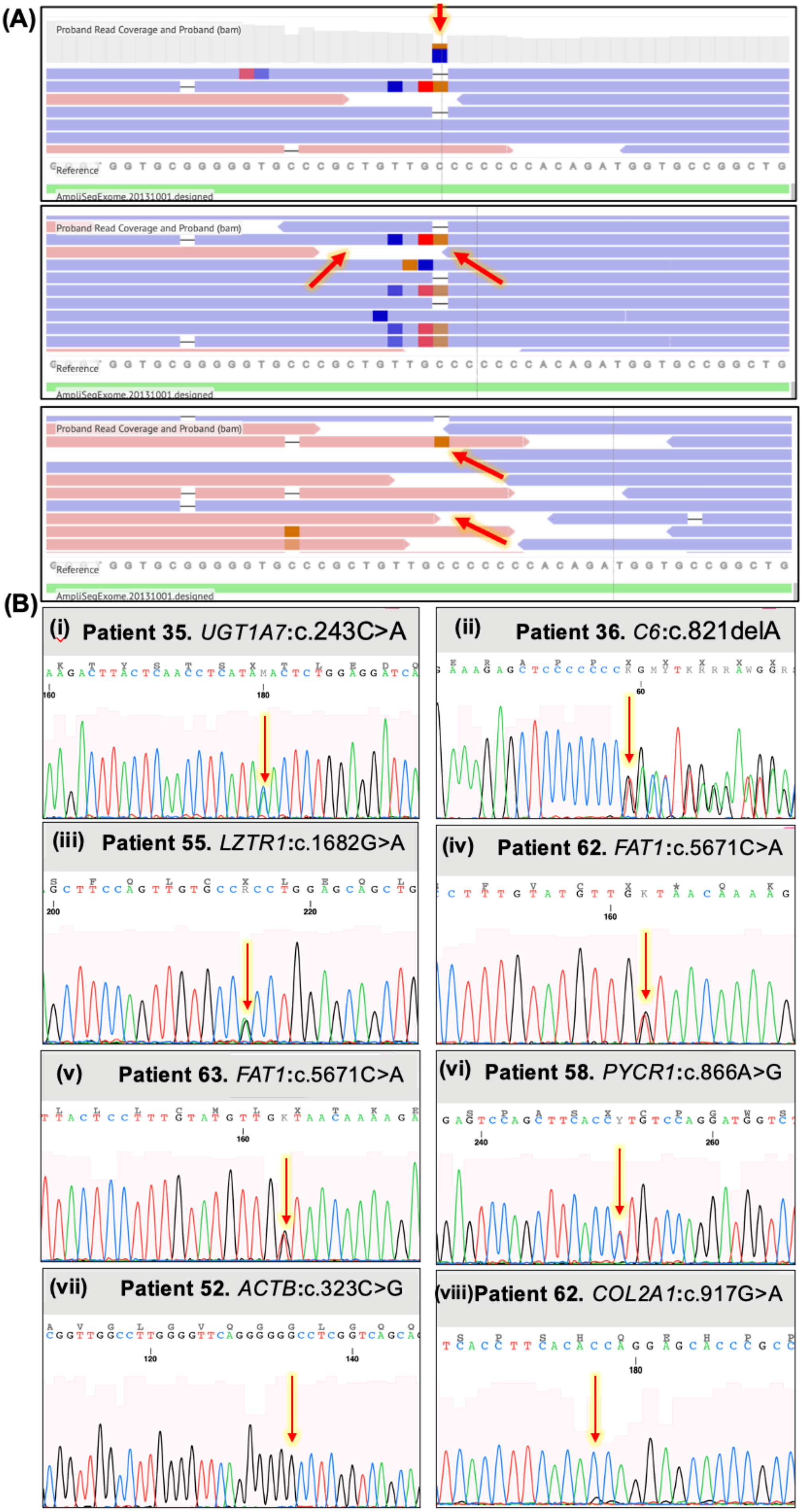
(A)
Proband Read Coverage and Proband (bam)
Reference
AmpliSeqExome.20131001.designed
(B)
(i) Patient 35. UGT1A7:c.243C>A
(ii) Patient 36. C6:c.821delA
(iii) Patient 55. LZTR1:c.1682G>A
(iv) Patient 62. FAT1:c.5671C>A
(v) Patient 63. FAT1:c.5671C>A
(vi) Patient 58. PYCR1:c.866A>G
(vii) Patient 52. ACTB:c.323C>G
(viii) Patient 62. COL2A1:c.917G>A

Supplementary Figure 1: Validation of the selected variants in patients with early-onset CRC. **(A)** Validation by IGV inspection: A picture of the *AXIN2*:c.1994delG variant in the exome data of patient 30 on the IGV software. The coverage and the position of the deletion and the substitution (top). The other reads at the same position with the ends of the amplicons of negative strand and the other variations around the position of the deletion, indicating an error-prone or artefact location (middle). The ends of amplicons on the forward strands with C to G change on the forward strand (bottom). **(B)** Chromatograms showing validation by Sanger sequencing with sequences at the variant's positions. (i), (ii) (iii), (iv), (v), and (vi) were validated. (vii) and (viii) were not validated resulting in exclusion.

(A)

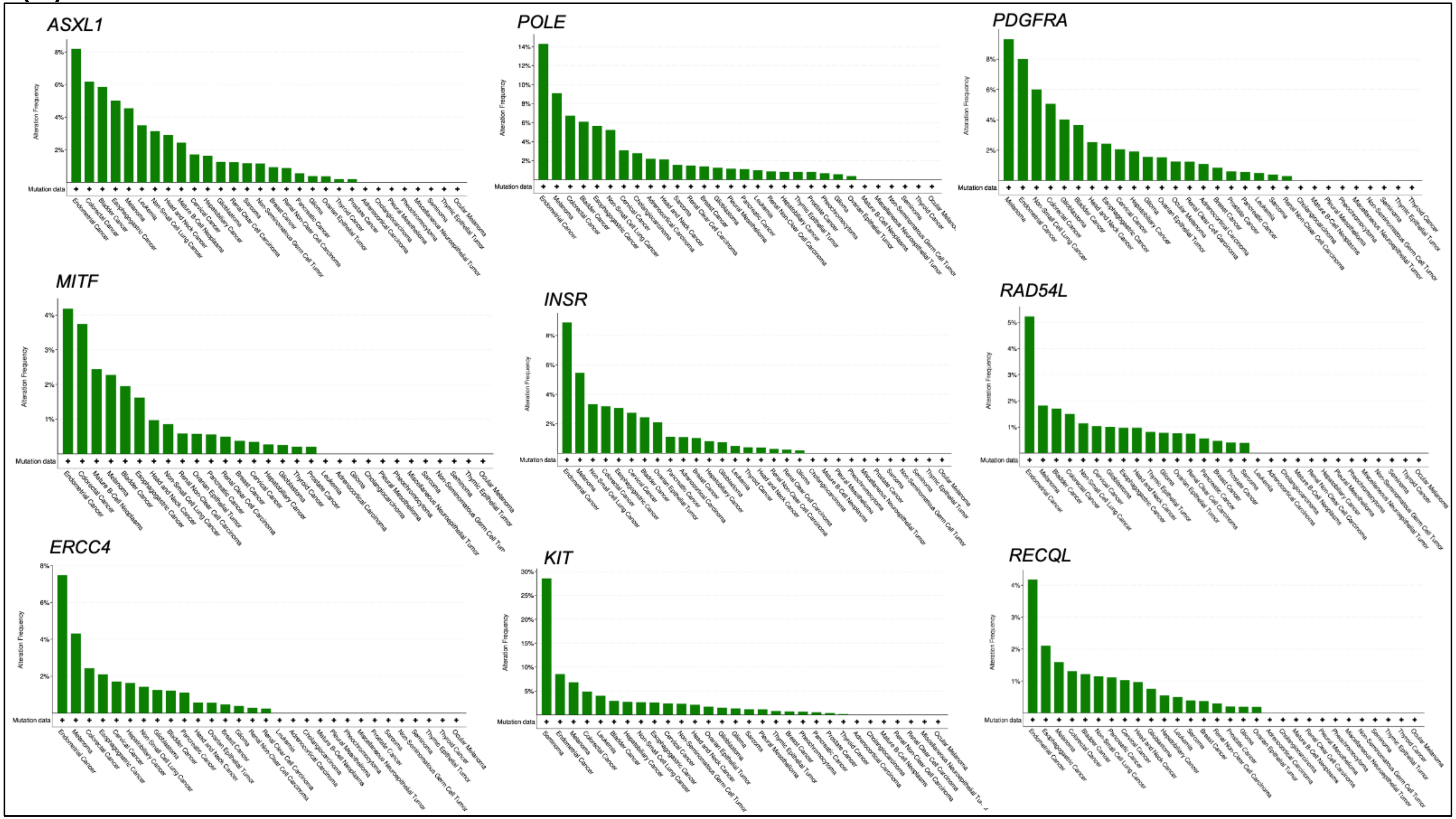

ASXL1
POLE
PDGFRA
MITF
INSR
RAD54L
ERCC4
KIT
RECQL
Alteration Frequency
Mutation data

(B)

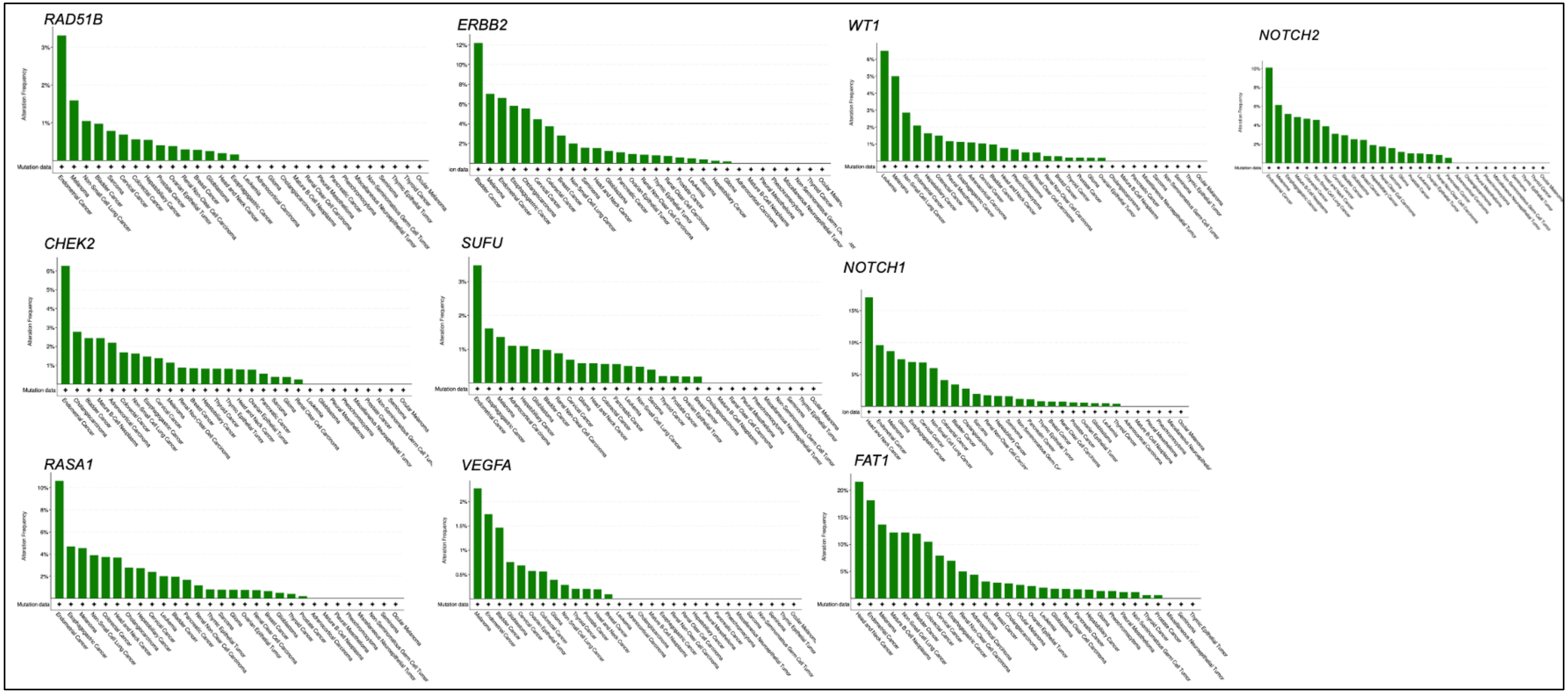

Supplementary Figure 2: Variant frequencies of genes of interest across different cancer types from TCGA PanCancer Atlas Datasets. **(A)** *ASXL1* exhibits the highest variant frequency in endometrial cancer and CRC. *POLE* shows the highest variant frequency in endometrial cancer and is notably present in CRC. *PDGFRA* is most frequently mutated in melanoma and endometrial cancer. *MITF* has significant variants in endometrial cancer and CRC melanoma. *INSR* displays high variant frequencies in endometrial cancer and melanoma. *RAD54L* shows the highest frequency in endometrial cancer. *ERCC4* has notable variant frequencies in endometrial cancer and melanoma. *KIT* variants were predominantly high in seminoma. *RECQL* is highly mutated in endometrial cancer. These findings provide insight into the diverse variant profiles of these genes across multiple cancer types, suggesting their potential contributions to oncogenesis. **(B)** Variant frequencies of genes of interest across different cancer types from TCGA PanCancer Atlas Datasets*; RAD51B, CHEK2, RASA1, ERBB2, SUFU, VEGFA, WT1, NOTCH1, FAT1, AND NOTCH2*.

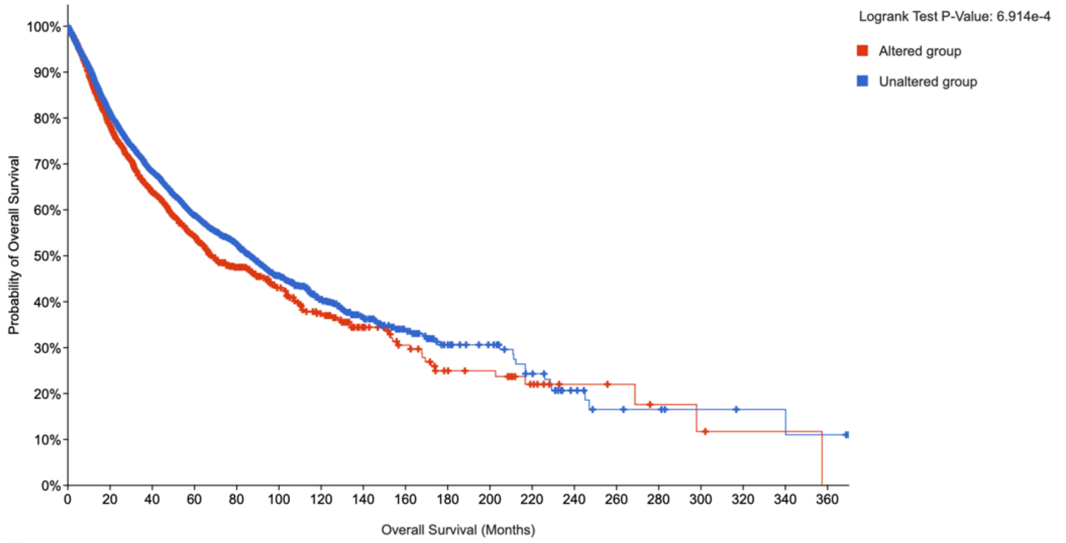

Number at risk (n)

	0	20	40	60	80	100	120	140	160	180	200	220	240	260	280	300	320	340	360
Altered group	2349	1302	684	383	219	127	86	54	36	23	20	12	6	5	3	2	1	1	0
Unaltered group	7945	4530	2275	1298	742	415	224	119	72	43	33	22	12	7	6	4	3	3	2

Survival plot summary

	Number of Cases, Total	Number of Events	Median Months Overall (95% CI)
Altered group	**2349**	**819**	**68.09 (62.79 - 86.76)**
Unaltered group	**7945**	**2398**	**86.30 (81.37 - 91.99)**

Supplementary Figure 3: Kaplan-Meier survival analysis from TCGA datasets comparing overall survival between lpVUS altered and unaltered groups. The altered

group, with variants in the genes containing lpVUS, demonstrated improved survival compared to the unaltered group.

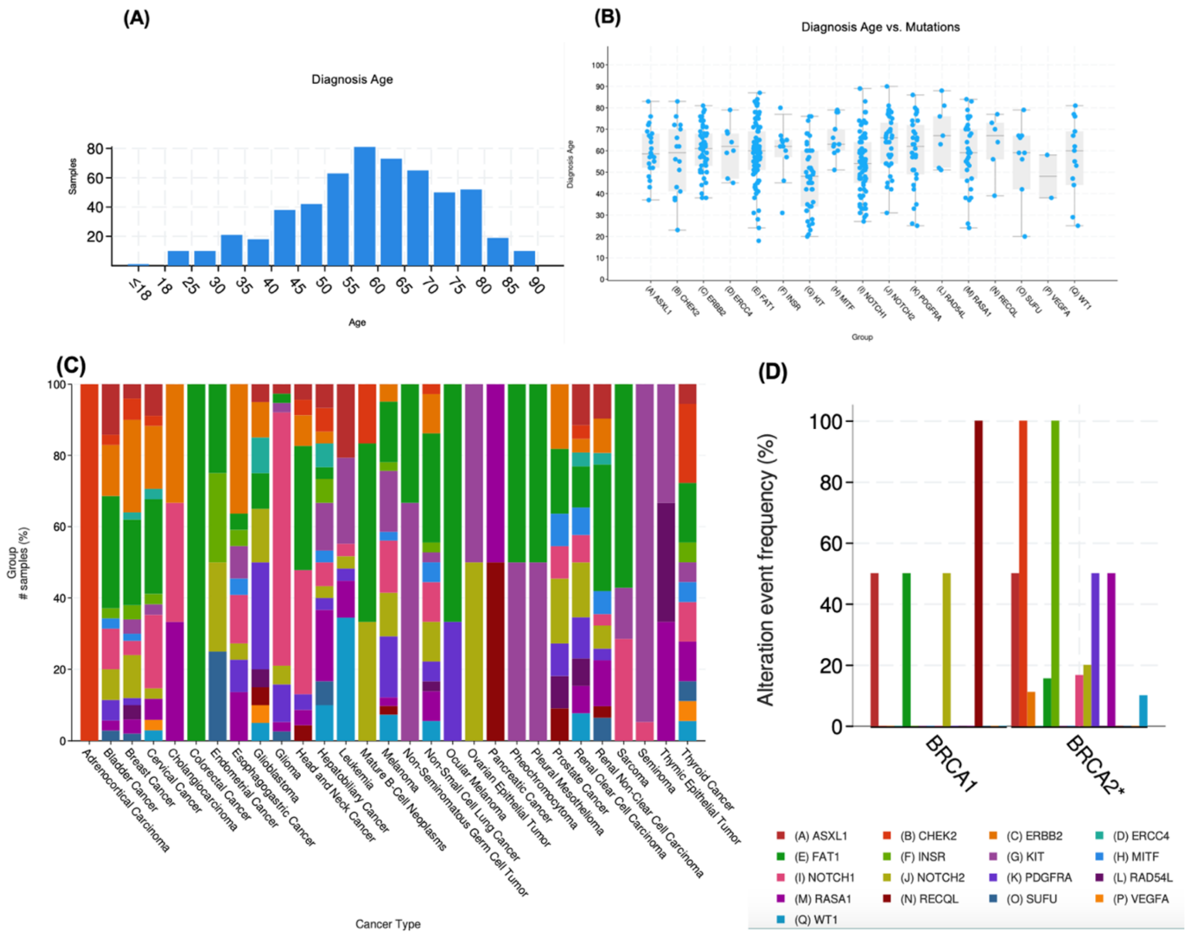

Supplementary Figure 4: Analysis of diagnosis age, variant distribution, cancer types, and alteration frequencies in selected genes. **(A)** Histogram displaying the distribution of diagnosis ages. **(B)** Scatter plot showing the relationship between age at diagnosis and the number of variants per sample. **(C)** Stacked bar chart depicting the proportion of samples with variants in specific genes across various cancer types. **(D)** Bar chart illustrating the frequency of alteration events in *BRCA1* and *BRCA2*, with the highest variant frequencies observed in *CHEK2* for *BRCA1* and *KIT* and *RAD54L* for *BRCA2*.
